# Dr. Mills on the Morbid Appearances Exhibited in Various Disorders of the Brain, on Dissection

**Published:** 1826-10-01

**Authors:** 


*An Account of the Morbid Appearances exhibited on Dissection in various Disorders of the Brain; with Pathological Observations, to which a Comparison of the Symptoms with the Morbid Changes has given rise.* By THOMAS MILLS, M.D. Licentiate of the King and Queen's College of Physicians. One vol. 8vo. pp. 239. London and Dublin, 1826.

WE have often remarked in this Journal, and indeed the truth of the remark is obvious, that there is but one way of arriving at correct information in the practice of physic—that of connecting the symptoms of diseases with their antecedent causes and post-mortem consequences—to which must be added a careful notation of the effects of remedies during life. Dr. Mills appears to have pursued this method for sixteen years past, and is now presenting the results to his professional brethren.

"My plan was, to note the symptoms and the effects of the remedies, and after death, the appearances presented on dissection.—At the dissection I was always accompanied by one, two, or three medical gentlemen, and it was my uniform practice to take down in writing, at the time, the account of the morbid changes from the lips of the examining surgeon:—this account, on my return home, I copied into my note-book, and then, the whole case being clearly before me, and my mind full of the subject, I wrote down the sentiments to which it gave birth.—My object was to correct any error into which I might have fallen with regard to the nature of the complaint and the mode of treatment, or, to satisfy myself of the propriety of the opinions I had previously entertained;—in short, my object was the discovery of truth.—The case, dissection and comment when finished, I laid by, without any view, at the time, to future publication." x.

Nothing can be more judicious than this method—nothing more meritorious than a faithful portraiture of the practice. If this example were generally followed, the science of medicine would ultimately attain all the certainty or perfection of which it is susceptible. No doubt, "the pursuit was laborious, the task irksome and painful," but Dr. Mills acquired thereby much valuable information—was enabled to detect errors—to correct prejudices. By this plan he flatters himself that he has been enabled to proceed from particulars to generals—and if he has not generalized too fast or too far, he has been fortunate indeed.

In this volume, which is the first of a series intended by Dr. Mills, several cases and dissections are detailed of hydrocephalus, cephalic fever, apoplexy, and epilepsy—in short, the whole volume consists of cases, with occasional short observations. Dr. M. remarks that the above diseases are considered distinct and independent, requiring a distinct and peculiar mode of treatment.

"Yet when we attentively examine the phenomena and course of each, when we compare them, one with the other, and witness the morbid appearances exhibited in all, and the effects of the same remedies, we shall be compelled to acknowledge that these diseases are closely allied, and, in the true spirit of philosophical research, be disposed to allow that the morbid actions which produce effects so alike, cannot, in their nature, be dissimilar."—*Pref.* i.

We admit, and so have some of our best pathologists, that there is a strong affinity between the diseases specified above—that many of the morbid processes, by which they are characterized, are of an analogous nature—and, consequently, that the prevention and treatment must hinge on similar principles in all. But we much doubt whether this consideration or admission will authorise us, in a pathological point of view, to attempt the identification of the tribe of diseases enumerated in this volume. We perceive too much disposition to this extensive generalization in the following passage.

"The phenomena of these diseases likewise merit the serious attention of the reader, in each he finds the prominent symptoms to be head-ach, delirium, stupor, coma, sense of weight or fulness of the head, vertigo, tinnitus aurium, convulsions, paralysis of the sphincters and other muscles, all of which are indicative of disordered actions of the vascular system of the brain, and of a disturbance of the sensorial functions.—To these symptoms we may add a pulse varying in strength, frequency, and regularity, a varying temperature of the skin and an irregular state of the secretions." xv.

This may be all true; but yet when we examine these diseases, not in their state of *greatest approximation*, but when they are in their ordinary or mean relation to each other, we shall find ample grounds, we imagine, for keeping them distinct on our nosological chart. Thus, let us look at two of them, which are considered by Dr. Mills as most nearly allied—apoplexy and epilepsy, and we shall be constrained to confess that, though they are sometimes combined, or run into each other, they are, in general, very different affections. Look at two men who fall down on the same floor, one in apoplexy, the other in epilepsy. In one, the muscular power is annihilated, in the other, it is so *supernatural*, we might almost say, that three or four strong men are required to control it. Surely these two most opposite conditions of the external phenomena would indicate something very different in the pathological state of the sensorium, on which the muscular power depends. In the one case, the paroxysm is over in a few minutes, or in half an hour, the patient falling into a sleep, and awaking unconscious of the accident. In the other, the patient dies in a day or two—or slowly recovers, with paralysis of some member—or perhaps comes out of the fit in a short time without any paralysis at all. In one case, the epileptic paroxysms will return, at various intervals, for years, without ultimately destroying life:—in apoplexy, it is very different:—one, two, or three attacks generally terminate the career of life, or deprive the patient of the use of one side of the body, and perhaps of intellect. In both cases do we employ the same remedial agents? What effect would the argentum nitratum, or the missletoe of the oak, have on apoplexy? Is the pathological condition of the brain the same in apoplexy and epilepsy? We much doubt it. The immediate cause of epilepsy is supposed, we know, by some to be a determination of blood to the brain, but this is not proved. It is more likely to be some peculiar *irritation* of the sensorium than a flow of blood. But in apoplexy we are certain that it is a flow of blood to the brain—and too often the extravasation of that fluid beyond the parietes of its vessels. These and many other reasons which we could easily urge, seem to suggest caution in amalgamating diseases which only occasionally assimilate in their pathological characters. It is now many years, indeed, since the elder Parry endeavoured to shew, in his Elements of Pathology, the *relationship* of the abovementioned and of several other diseases, but beyond this we think it is not safe nor correct to go, in the present state of our knowledge.

Of the immense collection of cases in this volume we can only select a comparatively small number for admission into our analysis, and these abbreviated according to our usual custom.

*Case* 1. A child, 2 years of age, had been ill about three weeks with bowel-complaint and fever, loss of appetite and spirits, heaviness of the head, and some intolerance of light—pulse 130 and tense—fæces greenish—moaning and sighing—rests its head on a chair or pillow. *Leeches to the temples—blister—purgatives—warm bath.* The child was relieved by these remedies; but the fæces were still green, and the pulse 120. On the second day the pulse had fallen to 90—the stools were still green—and there was stupor. On the 3d day, strabismus, convulsions, death.

*Dissection.* Vessels of the brain turgid—serous effusion between the membranes—three ounces of water in the lateral ventricles—six drachms of fluid in the pericardium—tubercles in the lungs and spleen—mesenteric glands enlarged—contents of the small intestines of a yellowish and greenish hue.

*Remarks.* This child had been supposed to labour under a bowel and bilious complaint, on account of the morbid condition of the fæces.

"During the illness the fæces were greenish; an appearance commonly imputed to the use of calomel, but which is more properly referrible to the condition of the bile; green bile was found in the gall-bladder and ducts, whence it was traced to the small and large intestines, every where tinging their contents with a green of a deeper or lighter shade, according to the quantity of bile present, and the quantity and nature of the matter with which it was mixed." 4.

We are glad to find, from this extract, that we are borne out by Dr. Mills, in the remark which we have repeatedly made respecting this green colour of the stools in children. A general opinion prevails that it is produced by calomel—whereas we have, times out of number, seen it occur and continue where no calomel had been used—nor are we convinced that when this appearance does occur, after calomel, it is the consequence of that remedy. Calomel will often dislodge morbid secretions from the bowels when other purgatives fail—and it has the peculiar effect of stimulating the liver and gall-bladder to discharge their accumulated secretions, and thus produce the phenomenon in a way very different from what is generally supposed. In the case above-detailed, there is unequivocal anatomical evidence of the existence of the green matter in various parts of the intestinal canal, and even in the gall-bladder and ducts. No calomel was given during Dr. Mills' attendance on the patient.

*Case* 2. This was a boy, seven years of age, who had been ailing for three months, with pains in his head and bowels, low fever, heaviness in the head, constipated bowels, and dark green motions. The mother of the boy laboured under scirrhous liver. When Dr. M. was consulted, the pulse was 126, skin cool, tongue white, complexion sallow—low delirium—sighing and moaning—cries out "Oh my head." Leeches—mercurial purgatives—blisters, and various means were used; but the boy ultimately sunk, with convulsions, *occasional loss of sight and hearing*, contracted pupils, paralysis, involuntary stools, and the usual phenomena of hydrocephalic effusion.

*On dissection*, water was found between the membranes, and no less than six ounces of this fluid in the ventricles. The liver was inflamed, and studded with tubercles, some of which were almost cartilaginous. The gall-bladder was full of green bile. The spleen was tuberculated, as was also the pancreas.

*Remarks.* We can hardly agree with Dr. Mills that, in this case, we have no clue as to which of the three organs (brain, spleen, or liver) was first affected. We think there can be very little doubt that the tuberculated condition of the abdominal viscera preceded, for a long time, the inflammation and effusion in the head. It is, therefore, probable, though against our author's opinion, that the morbid actions in these latter organs *did* stand in the relation of cause (whether predisponent or exciting) to the cerebral affection. There is a curious, though by no means uncommon circumstance related in the foregoing case, namely, the *occasional* recovery of sight and hearing, after these senses had been completely lost. This is one of the mysteries of the nervous system which we are not yet able to unravel.

We must pass over several cases of hydrocephalus, with only a slight allusion to a *lusus naturæ,* found in an infant seven days old. The child was fully formed and healthy at birth, but considerable emaciation took place on the fifth and sixth days, with jaundice, feeble pulse, coma, and vomiting. There was serous effusion in the brain, and the hepatic artery was entirely wanting. The liver was small and of a dark colour—the gall-bladder was also very small, and filled with a kind of substance resembling meconium. The fæces in the bowels were tinged with yellow bile.

Our author next presents us with a series of cases of hydrocephalus, consequent on other diseases, of which we shall be able to notice only one instance.

*Case* 3. Mr. K——, aged 42, of melancholic temperament, and subject to indigestion, laboured under inflammation of the liver, especially of the left lobe, with cough and præcordial oppression, which was cured by bleeding, blistering, and mercurial purging. During the preceding winter he had had cough, dyspnœa, pains in the chest, mucous expectoration, hectic fever, and uneasy feelings in the region of the liver. On the 23d January, 1822, he complained of pain and fulness in the head, confusion of ideas, want of rest, inappetency, irritability of mind, attended by frequent epistaxis, and obtuse pain in the liver; venesection and leeches relieved these symptoms, together with blisters to the head and mercurial aperients. 31st. The patient is cheerful, and talks of going down stairs. *Feb.* 1*st.* Sudden loss of speech, the sight and hearing remaining perfect, together with the power of deglutition. *Leeches to the temples—blisters to the legs.* He lingered till the 8th, when he expired.

*Dissection.* Surface of the brain highly vascular—serous effusion under the membranes—substance of the brain very firm—four ounces of water in the ventricles, and much serous effusion in the cellular structure about the optic nerves. In the thorax, there were adhesions, false membranes, and a pint of serous fluid in the right side. The right lung was completely hepatized, and contained several tubercles. The left was partially hepatized and tuberculated. The cavity of the pericardium contained some fluid, and this membrane was studded with minute tubercles, to an extent of three inches, with a corresponding state of the heart in apposition. The liver was tuberculated in a high degree.

*Remarks.* Hydrocephalus was doubtless the immediate cause of death in this case, and it is highly probable that this last disease was determined by the other visceral derangements.

Dr. Mills' next cases are those of recovery from supposed hydrocephalus. We quote the following passage, because it contains much truth, and explains what is meant by *recovery* from hydrocephalus.

"Hydrocephalus is, in every instance, a dangerous disease; insidious in its approach, and often rapid in its progress, effusion not unfrequently supervenes before the alarm is given; whenever, therefore, there is ground to suspect the existence of this disorder, when the head is engaged, though some of the symptoms be equivocal, a decided line of treatment should be pursued: now, as this complaint is inflammatory, or congestive in the *first* stage, and dropsical in the *second*, it is clear that general and local evacuants are indicated, and are the only remedies to be relied on; if these fail, effusion follows, when all our efforts too often prove abortive: in cases of recovery, therefore, hydrocephalus, correctly so called, can scarcely, in any instance, be said to exist; but there is inflammation or congestion of the vessels, threatening to terminate in that aqueous effusion from which it derives its name." 65.

We do not, indeed, suppose that recovery takes place after actual effusion—but we have every reason to believe that all, or almost all, the symptoms produced by effusion, will be produced by that turgescent state of the vessels which precedes effusion—and this state being removed, the apparent hydrocephalus is cured.

We shall here introduce the particulars of a case which is of frequent occurrence in practice, and leads to important considerations.

*Case* 4. *June* 2*d*. Master P———, ætat. 10, had been exposed to wet and cold, and then complained of fugitive pains in the forehead and occiput, languor, throbbing of the temples, alternations of colour in the face, bad appetite, and imperfect digestion. Pulse 110, skin hot. *Venesect. ad* ℥viij. *Five grains of calomel.* 3*d*. Temporary ease from the bleeding—blood sizy—three motions, yellow and greenish, with slime—urine high-coloured—head-ache and fever abated—moans and sighs occasionally. *Leeches to the temples—four grains of calomel—an aperient draught.* 4*th*. Much relieved by the leeches—the calomel has brought away fæces like tar—urine turbid—skin pale, yet pungent to the touch—pulse 120—uneasiness in the right hypochondrium—tongue furred. *Twelve leeches to the temples—calomel repeated.* 5*th.* High fever, and throbbing of the temples—pulse 130—fæces dark and olive-coloured—tongue parched and brownish—uneasiness in the region of the liver abated. *Venesectio ad* ℥viij.—*cathartic—warm bath.* 6*th.* The blood drawn was dense—temporary relief, but the symptoms became exasperated *last* evening. *Fifteen leeches to the temples—blister to the nucha—cathartic—warm bath.* 7*th.* Intolerance of light and noise—stupor—delirium—pulse irregular—skin hot and dry—fæces black. *Venesectio ad* ℥vj. *Balneum tepidum—cathartic—anodyne in the evening.* 8*th.* Better this morning—exacerbation in the evening. *Cathartic.* 10*th.* Return of head-ache and fever—restlessness, moaning, and sighing—fæces yellow, urine turbid. *Twelve leeches to the temples—three grains of calomel and half a grain of the watery extract of opium every three hours.* 13*th.* Head-ache and fever considerably abated; but still there are morning remissions and evening exacerbations—some return of appetite. *The calomel and opium continued.* 16*th.* Gradual amendment—bowels opened by simple aperients—gums are sore from the mercury. *The calomel and opium continued.* 20*th.* Some ptyalism—skin moist and warm—free from head-ache and fever—return of appetite. Convalescent.

*Remarks.* We consider the foregoing case as a very interesting one, where the most active and judicious treatment was put in force, and where the fatal effusion was, in all human probability, warded off by the skill of the physician. We shall give our author's own commentary on the case, as we fully coincide in his sentiments on this occasion.

"This was a case of threatening hydrocephalus, accompanied by a considerable derangement of the hepatic functions,—the shooting pains in the head, the languor, the intolerance of light and noise, the moaning and sighing, indicated a morbid state of the vessels of the brain, while, from the appearance of the excretions and the uneasiness or pain in the right hypochondre, it was clear that the functions of the liver were much disturbed.

"It is not unusual to find these two organs simultaneously diseased—in some cases the brain is primarily engaged, in others, the liver; and, how far they stand in the relation of cause and effect, it is not, at all times, easy to determine; one thing, however, is clear, that if either viscus has been long disordered in its actions, or has been altered in its structure, the other, from their mutual relations and sympathies, must suffer from the derangement; this fact is well known to every practitioner: when, therefore, a predisposition to hydrocephalus exists, it is rational to infer that a morbid condition of the liver will induce congestion or excitement of the brain, and finally, effusion into its cavities.

"The present case affords an example of the advantages to be derived from the prompt employment of active measures; indeed, when danger is present, the loss of a day, or of an hour, may be the loss of life.—Here we may notice the good effects of calomel and opium after depletion,—these remedies combined are often found to allay irritation and procure rest; moreover, they sometimes succeed in equalizing the circulation, by causing a determination of blood to the surface." 71.

Before taking leave of the subject of hydrocephalus, we are induced to give the particulars of one more case, the importance of which will be presently seen in the commentary.

*Case* 5. *October* 20. Master E——, ætat. 9, was seized a fortnight ago, without obvious cause, with head-ache, nausea, and loss of appetite, followed by pyrexia. *Leeches had been applied to the temples*, and *mercurial and saline aperients exhibited*, but without relief. The pain of head is now acute—temples throbbing—face flushed—eyes suffused—pulse 124 and strong—skin hot and dry—tongue foul—bowels open—fæces green and yellow—urine high-coloured. *Venes. ad* ℥viij. *calomel and pulv. Jacobi—enem. purg.* 21. Blood buffed and dense—temporary relief from the bleeding—delirium—restlessness—green and yellow fæces—pulse 130—head-affection still violent. 20 *leeches to the temples. The other medicines to be continued.* 22. Temporary relief as before, but all the symptoms now as violent as ever. Wild expression of the countenance. *Venesect.* ℥viij.—*blister—same medicines.* 23. Moaning, screaming, acute head-ache, alternate pallor and flushing. Green fæces passed involuntarily. 15 *leeches to the temples—calomel and James's powder continued—blisters to the head.* 24. No alleviation of the symptoms—pupil of one eye contracted, the other dilated—both sensible to light. *Blister to the fore-head. Medicines continued.* 25. Pulse irregular, tense, and quick—moans, and screams—" Oh my head!"—involuntary dejections. *Same medicines.* 26. High delirium—jactitation—sight of left eye lost—pupil of right eye dilated—pulse slow and intermitting—moaning and sighing—involuntary dejections—convulsions of the extremities. *Sixteen leeches to the temples—blister to the nucha—calomel and pulv. Jacobi continued.* 27. Great tossing of the head—sight of right eye almost lost—vacant expression of countenance—body convulsed. *Blister to the vertex. Medicines continued.* 28. Deglutition difficult—involuntary dejections—tossing and rolling of the head—moaning—pulse frequent—loss of speech. 29th and 30th. Same state.

*November* 3. Considerable flow of urine—more equable temperature of skin—expression of countenance wild and sometimes idiotic—speech indistinct. *November* 6. Some alleviation of symptoms—fæces yellow and green—urine copious—pulse more regular and soft—pupils more sensible. From this time there was a gradual improvement, although a gangrenous spot occurred on one hip, which retarded recovery. On the 12th January, he was still confined on account of the sores, but the cerebral affection was entirely gone.

*Remarks.* This case exemplifies the observation we made respecting the hydrocephalic symptoms depending on pressure of blood, not effusion of water. Desperate as were the phenomena in this instance, still we do not attribute them to actual effusion—at least of any extent, but to an inflamed and congested state of the cerebral vessels, which was ultimately conquered by bold and persevering measures. Dr. Mills, however, is of opinion that there was actual effusion in this case, and we give his remarks on the occasion.

"Most, if not all the symptoms which are considered diagnostic of hydrocephalus, in its advanced stage, were here present—every sense was blunted, the power of vision and hearing was suspended for several days,—deglutition was imperfect,—the speech was indistinct, the sphincters were relaxed—there were sighing, moaning and screaming, and, finally, convulsions, yet the patient recovered; indeed, so hopeless did the case appear to me, that for two or three days I gave up my attendance. Cases of this kind are rare, and therefore valuable; they teach us, never while life remains, to desert a patient, and never, to be too decided in our prognosis.—Life may be continued for years with effusion in the brain, as it may with water in the thorax or abdomen, but, there is this difference,—recovery occasionally takes place in the latter disorders, but when the brain is thus affected, very rarely indeed.—To what, in the present and similar instances of recovery, are we to ascribe the favourable result? Is it not to the action of the absorbent vessels of the brain? And, as every other organ of life is furnished with its complete system of vessels—of arteries, veins, absorbents, &c. can we suppose that the brain, an organ of such magnitude, and of such vast importance in the economy of the human frame, forms an exception to this law of nature?

"About the period of the incipient amendment in this child, the flow of urine was considerable, from which we may infer, that the secretory and absorbent vessels of the system were called into action, and that the absorbents of the brain partaking of this activity, the fluid effused was gradually absorbed." 95.

We must pass over a vast collection of cases and dissections of cephalic fever and of apoplexy, which fill a large portion of the volume under review, and which are valuable records for reference, in order to dwell a little on the subject of epilepsy, the pathology of which is not so well understood as that of apoplexy.

EPILEPSY.

*Case* 6. *February* 11, 1822. At three months old, Miss B. was attacked with epileptic convulsions, accompanied by pyrexia, and throbbing of the temples. Such attacks recurred at irregular intervals, for eight or nine months, when she was weaned, and sent into the country, where she resided five years. During this period the fits seldom recurred, but the little patient became rickety, and, at times, idiotic.

The head was enlarged and the belly tumid—the limbs were feeble and emaciated. In the course of the next five years, great attention was paid to her health, and some amendment took place, both in body and mind; but epileptic seizures still occasionally recurred. During the last six years of her life, she was afflicted with head-aches, and was subject to stupor, coma, vertigo, and tinnitus aurium, together with despondency and irritability of mind. The epileptic fits continued. At the age of 15 she menstruated, and died in a fit of apoplexy in her 16th year. The day before her death she dined with a good appetite—was able to walk about, and converse with her family.

"*Dissection.* On removing the cranium, numerous red points present themselves from torn vessels on the outer surface of the dura mater, the veins on the surface of the brain are turgid.—Between the dura mater and Arachnoid membrane is a watery fluid; in some places the Arachnoid membrane is opaque.—The falx is adherent to the lateral hemispheres, and these to the anterior lobes of the brain. The septum lucidum is destroyed, with the exception of a few shreds, which had been vessels.—The anterior part of the fornix is nearly removed by absorption.—The foramen monroianum is so enlarged as to allow the thumb to pass from one lateral ventricle into the other.—The lateral ventricles contain one pint by measure of a watery fluid; the appearance of this cavity in the substance is truly frightful.—The third ventricle is so much enlarged as to admit the fore-finger.—The iter à tertio ad quartum ventriculum will admit a goose quill.—The substance of the brain is considerably reduced, within the ventricles, it is of the consistence and feel of leather.—The optic nerves are smaller than usual. —The cranium is enlarged, and its bones, in some parts, are thinner than natural." 213.

The foregoing case is worthy of record, on account of the vast collection of water—the absorption or expansion of the brain—and the preservation of a considerable degree of intellect, under such terrible circumstances. There can be little doubt, however, that the hydrocephalus had been going on almost from birth. We knew of an instance where a large quantity formed in four years in the head of an adult, after an attack of apoplexy, and where the patient could walk about, eat, sleep, and converse till the moment in which he fell down in a fit and expired.

*Case* 7. This was a gentleman of temperate and sedentary habits, melancholic temperament, delicate frame, and subject to indigestion. He laboured under epilepsy eight years, the attacks varying in frequency and violence. They were preceded by pain and heaviness of the head, stupor, confusion of ideas, imperfect vision and tinnitus aurium. They were followed by stupor, melancholy, and loss of memory. On the three days preceding his death, he had three successive fits, after the first of which there was paralysis of the right side—after the second, dilatation of the pupils with loss of vision——and the third was succeeded by violent convulsions and death.

" *Dissection.* The dura mater is thickened in several places.—A gelatinous fluid of a pale white is generally diffused between the Arachnoid membrane and pia mater.—The vessels on the surface of the cerebrum are turgid with blood of a dark colour.—On cutting through its substance it is found preternaturally hard and dotted with numberless dark-coloured specks.—The septum lucidum is strong and thickened.—The cerebellum presents the same diseased appearance as the cerebrum.

" The colon is distended with flatus.—The mucous coat of the small intestines is dotted with numerous dark spots apparently arising from venous congestion.—The bladder is unusually large and distended with urine.—The liver is considerably enlarged and hardened, and firmly adherent to the diaphragm ;—on cutting into its substance it appears much paler than natural." 216.

Our author has observed this state of the brain in other epileptics, and has thence been led, in every case of this complaint, accompanied by diseased cerebral action, to establish a drain from the vertex, and sometimes from the nucha. In the majority of cases, the remedy was not successful, but it " generally mitigated the violence of the paroxysms, and, in a few instances, has apparently cured the disease."—Such was the following instance.

*Case* 8. Mrs. C———, ætat. 46, had been subject to epileptic fits for four years, which returned at irregular intervals, but most frequently in summer. They were often violent, and continued with some intermissions for one or more days. These fits are brought on by troubles of mind, constipation of bowels, or bodily fatigue—and sometimes occur without any apparent cause. The attack is always preceded by vertigo, pain or sense of fulness in the head, confusion of ideas, or tinnitus aurium. Very often the digestive organs are deranged for some days preceding the attack, as evinced by a painful distention of the stomach and bowels, loss of appetite, offensive eructations, and furred tongue. The lady has been subject to hepatic derangement and despondency of mind for some time.

Blood-letting had been generally employed in the paroxysms, followed by mercurial purgatives, &c. Dr. Mills directed a drain to be established on the vertex by antimonial ointment—and four grains of James's powder with two of calomel to be taken every night, followed by a moderate dose of Epsom salts the next morning. *This was on the 6th July.* By the 30th of the same month, the gums were sore, a copious discharge was established from the vertex, and there had been no return of the fits. This immunity lasted till the 12th October, when she was threatened with a paroxysm, but the attack was warded off by venesection and a brisk purgative. The drain was continued, and she went on till the 18th July of the succeeding year, without any paroxysm. She then discontinued medicines, but kept the drain open. She was taken leave of as cured.

*Remarks.* "The case is instructive; it shews that much benefit may occasionally be derived from relieving the brain when this organ is the seat of the disease.—It likewise shows that the antimonial ointment properly employed, may produce, for months, a copious purulent discharge from the vertex, and thus may prevent a recurrence of an epileptic paroxysm.—This patient thought she experienced considerable relief from the use of James's Powder; and from its action on the cutaneous vessels, no doubt it contributed to equalize the circulation, and thus to diminish or prevent congestion of the brain.—It may be proper to mention, that on one occasion, an approaching fit was prevented by a removal from the town into the country, and by the operation of a brisk cathartic." 223.

By a note appended to this case, it appears that this lady is still subject to attacks, at intervals of four or six months, but the fits are slighter than formerly. She has again established the discharge from the vertex.

We can only advert to one more case before we close our notice of this volume.

*Case* 9. Mr. B——, aged 36, addicted to intemperance, was attacked, after a fit of intoxication, with violent convulsions of the head, face, tongue, body, and extremities. These were followed by stupor and insensibility—pulse 116, strong and intermitting—skin hot and moist—tongue foul and yellowish—face flushed—bowels constipated. *Bled and purged.* Next day, there was delirium, and a return of the convulsions. Bled from the temporal artery, and again purged. He gradually got better, and in ten days was pronounced convalescent. About six weeks after this, on drinking half a pint of spirituous liquor, he lost, for several minutes, all sense and power of motion, followed by convulsions of body and extremities, foaming at the mouth, distortion of the face, &c. He was *bled and purged*—and the next day *leeched*, the same paroxysm having returned. Delirium and other bad symptoms now came on, and he died on the third day. We shall give the dissection in the words of our author, premising that this patient, during the preceding year had laboured under repeated attacks of maniacal delirium, the consequence of intoxication.

"The os frontis is of unusual thickness, the arachnoid membrane is raised from a quarter to half an inch from the pia mater by a serous effusion, which extends over the entire surface of the cerebrum.—A considerable quantity of serous fluid escaped on cutting into the right ventricle; the left and third ventricles are distended with the same fluid.—The walls of the lateral ventricles are considerably firmer than natural.—Plexus Choroides very pale.—The quantity of serous fluid found upon the surface of the brain, in the ventricles and the base of the cranium, may be estimated at about six ounces.—The liver is larger and harder than usual; externally it is of a brick-colour, and when cut into tubercles or marks of inflammation are discoverable.—The gall-bladder is distended with bile of a greenish-yellow.—The spleen is paler, smaller, and softer than natural; it yields to the pressure of the hand, and when thus broken resembles coagulated blood.—The omentum is loaded with fat.—The coats of the stomach are thickened, on the mucous surface are observed red and purple-coloured patches differing in their size and figure.—The mezenteric glands are loaded with fat.—The mucous coat of the ilium in some places is highly vascular.—The lungs contain more air than usual; in the superior part of the left lung are three portions of calculous matter, each about the size of a small pea.—The heart is fatty but natural.—The pericardium contains about a dram and a half of serous fluid." 229.

The foregoing case is interesting, as shewing the melancholy effects of intemperance, especially in spirituous liquors, on the great organs of the body, including the organ of mind!

We must now take our leave of Dr. Mills' work, hoping that we have presented fair specimens of the matter and manner of the publication, while, at the same time, we have enriched our pages with some valuable and authentic facts that are calculated to guide the judgment and excite the attention of the practitioner. We shall be happy to pay our respects to the forthcoming volume from the same pen, on diseases of the chest. We think the zeal and candour of our author are highly praise-worthy, and entitle his work to a favourable reception from his brethren on both sides of the channel.

